# Characteristics of Relative Age Effects and Anthropometric Data in Japanese Recreational and Elite Male Junior Baseball Players

**DOI:** 10.1186/s40798-018-0165-9

**Published:** 2018-11-30

**Authors:** Yoichi Katsumata, Kohei Omuro, Naotoshi Mitsukawa, Hiroki Nakata

**Affiliations:** 1 Faculty of Applied Bio-Science, Tokyo University of Agriculture, 1-1-1 Sakuragaoka, Setagaya-ku, Tokyo, 156-8502 Japan; 2 Center for Liberal Arts and Sciences, Hachinohe Institute of Technology, 88-1 Obiraki, Myo, Hachinohe, Aomori, 031-8501 Japan; 3 Faculty of Human Sciences, Toyo Gakuen University, 1-26-3 Hongo, Bunkyo-ku, Tokyo, 113-0033 Japan; 4 Faculty of Human Life and Environment, Nara Women’s University, Kitauoya-Nishimachi, Nara, 630-8506 Japan

**Keywords:** RAE, Sport, Japan, Adolescent, Anthropometry

## Abstract

**Background:**

The mechanisms underlying the relative age effect (RAE), a biased distribution of birth dates, in sport events have been investigated for more than two decades. The present study investigated the characteristics of the RAE in baseball and anthropometric data (height and weight) among recreational junior baseball players as well as elite players, using data extracted from national surveys.

**Methods:**

Birth and anthropometric data were obtained from 4464 Japanese students as elementary school, junior high school, and high school players.

**Results:**

Significant RAEs were noted in recreational junior high school and high school players, but not in elementary school players, and the effect size became larger with increasing grade (0.063 in elementary school players, 0.151 in junior high school players, and 0.227 in high school players). We also observed a significant RAE for elite elementary, junior high, and high school players. However, the effect size of elite players became smaller with increasing grade (0.563 in elementary school players, 0.358 in junior high school players, and 0.343 in high school players). Height and weight values were significantly larger in elite elementary and junior high school players than in recreational elementary and junior high school players, but height was almost the same between recreational and elite high school players.

**Conclusion:**

The characteristics of RAEs differed between recreational and elite junior baseball players. Anthropometric data were also different, depending on birth data, and between recreational and elite baseball players in elementary and junior high school students.

**Electronic supplementary material:**

The online version of this article (10.1186/s40798-018-0165-9) contains supplementary material, which is available to authorized users.

## Key Points

- The present results indicate that the patterning of RAE differs between recreational and elite junior baseball players.
- Biological maturity occurs earlier in elite elementary and junior high school players than in recreational elementary and junior high school players.
- The height was almost the same between recreational and elite high school players, indicating that the status of biological maturity evens out in high school.

## Background

The “relative age effect” (RAE) has been investigated as one of the additional factors to become an athlete, since relatively older players (children) within the same age group have an advantage over relatively younger players (children). Within the same age category, a difference of almost 1 year might exist between the oldest and youngest players. Numerous previous studies reported that RAE bias exists in many sports, including soccer [1, 2], tennis [3], cricket [4], basketball [5], handball [6], swimming [7], track and field [8], sumo wrestling [9], rugby [10], judo [11], ice hockey [12], and alpine ski racing [13]. The attributes of greater height, mass, aerobic power, muscular strength, endurance, and speed provide performance advantages in most sports [14, 15], giving relatively older children advantages in sporting ability, psychological confidence, instruction, and playing time. On the other hand, some studies reported an advantage for relatively younger gymnasts and jockeys [9, 16], suggesting that late puberty in these sports is an advantage for selection. To date, a variety of sports contexts differing in age categories, levels of competition, and cultures have been assessed to exam RAEs (see a meta-analytical review, [15]). For example, RAEs are more prevalent in male sports, when the sport is very popular and there is strong competitive selection for teams and competition [8].

In addition to many kinds of sports, several studies have reported the existence of significant RAEs in major league baseball (MLB) players [17, 18] and professional Japanese baseball (Nippon Professional Baseball (NPB)) players [9, 18]. Moreover, some historical analyses were performed to clarify the beginning of RAEs in a country and compare differences in the skew of RAEs among generations, based on socio-cultural factors. Grondin and Koren [18] focused on RAEs over the history of MLB. They found no significant RAE among players born in the 1920s and 1930s, but a significant effect among those born in the 1940s, 1950s, and 1960s. Abel and colleagues [19], who used an online database, found that the distribution of birth months was significant for male but not female baseball players born in 1914–1938. Nakata and Sakamoto [20] reported a statistically biased distribution of birth dates among NPB players born in the 1940s and onward, and a strong tendency of significant RAEs was observed among players born in the 1910s, 1920s, and 1930s. These studies suggest that the magnitude of RAEs changed with time, and that socio-cultural factors, such as international competitions, and media coverage may have markedly contributed to this. To date, two prospective analyses of NPB players have been reported. Nakata and Sakamoto [21] investigated the association of RAEs with a high school vs. university background in Japanese professional soccer and baseball players. They showed significant RAEs among professional players who graduated from university or college. This finding suggests that long-term disadvantages exist, even if physical maturity evens out between relatively older and younger players toward adulthood. Nakata [22] also reported that RAEs among NPB players were associated with the lengths of professional careers, indicating long-term effects after becoming professional players.

These previous studies were based on the number of professional baseball players; however, no data on junior baseball players were presented. Baseball was not an official event at the London Olympics 2012 or Rio de Janeiro Olympics 2016. This may be related to baseball not being a global sport, but it will be an official event at the Tokyo Olympics 2020. Many previous studies examining RAEs in other sports, which have been official events at the Olympics or World-Cup, have focused on their existence, the mechanisms, and solution in junior players. Just like other sports, in the near future, marked popularity and a strong competitive selection in baseball for teams and competition will occur globally. In order to reduce the selection bias and resolve RAEs preliminarily, the phenomenon of the RAE on junior baseball players should be investigated. Thus, the aim of the present study was to investigate the characteristics of RAEs among recreational samples of junior baseball players as well as elite players in Japan. Some previous studies reported that the skew of RAEs was related to players’ level and categories [2, 3, 10, 23–25]. Japan has applied a unique annual-age grouping for education since 1886, basing group assignment on student’ birthdates between April 1 (the “new” year) and March 31 of the following year among elementary, junior high, senior high, and university (college) students and in government and company employment. Sports calendars also follow this system, giving Japanese children and adolescents born between April and June a relative age advantage over those born between January and March. We divided recreational and elite Japanese junior baseball players into three categories: elementary school, junior high school, and high school.

In addition, we examined the relationship between RAEs and anthropometry in recreational and elite junior baseball players, and compared differences in anthropometric data between recreational and elite junior baseball players. A previous study investigated RAE-related differences in anthropometric and physical fitness data of a general sample of Japanese elementary and junior high school students [26]. This study observed a significant RAE for boys aged 7 to 15 years and for girls aged 7 to 10 years on both anthropometric and fitness data. Based on this study, the current study analyzed the relationship between RAEs and anthropometric data in junior baseball players.

We also compared these data with NPB and MLB players in order to clarify the characteristics of RAEs in Japanese junior players. National teams of Japan and the USA are at the highest level in baseball. Japan won the 1st and 2nd World Baseball Classic in 2006 and 2009, while the USA won the 4th World Baseball Classic in 2017. Thus, we considered that the research on the RAE on professional players in these two countries might help to understand the RAE mechanisms in baseball. Almost 20 years ago, Grondin and Koren [18] reported differences in the skew of RAEs between MLB and NPB players. We confirmed whether this difference remained unchanged.

## Method

### Samples

In the Japanese school system, the school year in Japan begins in April, and school grades are defined as 6 years of elementary school (7–12 years old), 3 years of junior high school (13–15 years old), and 3 years of high school (16–18 years old).

Data on the birth date, height, and weight of players were obtained by a questionnaire. Data on recreational elementary school players (*N* = 844 males) were obtained from national surveys in 2014 and 2015. Recreational players were defined as those who played for school teams at the local level. Data on elite elementary school players (*N* = 157 males) who played in a national elementary school tournament were also obtained from the surveys in 2014 and 2015. Elite players were defined as those who played for school teams that participated in national championship games. The definition of recreational and elite was the same for the junior high and high school players. The age of elementary school players was 9–12 years old. The survey included some questions on sex, height, weight, birth date, brothers configuration, baseball position (pitcher, catcher, infielder, and outfielder), baseball experience (month), play level (national, prefectural, and local), and future plans. The real survey (in Japanese) is put on the Web as Additional file 1. We sent surveys to the Japan Rubber baseball Association.

Data on recreational junior high school players (*N* = 1426 males) were observed from the national surveys in 2015–2017. We sent surveys to approximately 2000 junior high schools. Data on elite junior high school players (*N* = 450 males) who played in a national junior high school tournament were obtained from the survey in 2015. We sent surveys to 27 junior high schools that had participated in national championship games. The age of junior high school players was 13–15 years old.

Data on recreational high school players (*N* = 830 males) were also obtained from the national survey in 2015. We sent surveys to approximately 4000 high schools. Data on elite high school players (*N* = 757 males) who played in a national high school tournament were obtained from the survey in 2015. We sent surveys to 32 high schools that had participated in national championship games. The age of elementary school players was 16–18 years old.

In the present study, we focused on only male junior players, because the number of female junior players was small. For example, there are only 26 high schools with a female baseball team in Japan (http://www.girls-baseball.jp/), while approximately 4000 high schools have a male baseball team.

Data on professional Japanese baseball players (*N* =  2238 males) who played in NPB from 1984 to 2015 were extracted from an official website (http://npb.jp/) and an official publication [27]. The age of all NPB players was over 19 years old. For comparison, data on MLB players (*N* = 992 males) were also obtained in 2015 from an official website (https://www.mlb.com/). The age of all MLB players was over 19 years old.

The study was approved by the Ethics Committee of Tokyo University of Agriculture, Tokyo, Japan. Informed consent and assent were sought prior to the study. Consent was sought from the participants and/or their caregivers. This study was performed in accordance with the standards of ethics outlined in the Declaration of Helsinki.

### Data Analysis

Japanese junior baseball and professional players were divided into four groups based on their month of birth: Q1 (April–June), Q2 (July–September), Q3 (October–December), and Q4 (January–March of the following year). Foreign players in the Japanese professional baseball leagues were excluded because they had not passed through the Japanese school system. Baseball players in MLB were divided into four groups based on their month of birth: Q1 (August–October), Q2 (November–January), Q3 (February–April), and Q4 (May–July), because the cutoff date for junior baseball league in the USA was August [16, 17]. Chi-square tests were conducted on the birth dates in each group according to the four quarters to determine the significance of deviations for the expected number of births in each quarter, assuming an equal distribution (e.g., *N* = 100, expected quartile count = 100/4 = 25). National birth statistics for males in Japan show almost 25% in each quarter since the 1960s (Ministry of Internal Affairs and Communications, the Statistics Bureau and the Director-General for Policy Planning of Japan: http://www.soumu.go.jp/) [20]. The effect size of chi-square tests was also calculated in each group. The odds ratio (OR) and 95% confidence intervals were then calculated to provide additional information for both quartile and half-year distributions, following a previous review article [15]. The ORs for the Q1, Q2, and Q3 vs. Q4 comparisons were interpreted as follows: OR < 1.22, 1.22 ≤ OR < 1.86, 1.86 ≤ OR < 3.00, and OR ≥ 3.00, indicating negligible, small, medium, and large effects, respectively [25]. In addition, data on the height and weight were separately subjected to the Kruskal-Wallis test to clarify differences among Q1–Q4 in each group as a non-parametric test, since the assumption of a normal distribution for all data was violated. The data for height and weight were also separately subjected to the Mann-Whitney *U* test to compare differences between recreational and elite groups in each quarter as a non-parametric test. Statistical tests were performed using computer software (SPSS for windows ver. 22.0, SPSS). Significance was set at *p* < 0.05, and the *p* values are shown as *p* < 0.05, *p* < 0.01, and *p* < 0.001.

## Results

Table 1 presents the results of chi-square tests for each group. Significant RAEs were observed in the elementary, junior high, and high school players (*p* < 0.001, respectively). In addition, significant RAEs were observed in the NPB players (*p* < 0.001) and MLB (*p* < 0.05). Table 2 demonstrates OR and 95% confidence intervals for each group. The larger effects of OR were observed in Q1, and Q2 vs. Q4 among high school players (OR = 2.06, and 1.88, respectively), and among NPB players (OR = 2.20, and 1.93, respectively).

**Table 1:** Distribution of all players divided into quartiles in each group

	Q1		Q2		Q3		Q4		Total	*X* ^2^	Effect size
	*n*	(%)	*n*	(%)	*n*	(%)	*n*	(%)	*n*		*w*
Elementary school	298	29.8	259	25.9	244	24.4	200	20.0	1001	19.27***	0.139
[Predicted]	251		250		250		250				
Junior high school	595	31.7	513	27.3	420	22.4	348	18.6	1876	74.32***	0.199
[Predicted]	469		469		469		469				
High school	529	33.3	483	30.4	318	20.0	257	16.2	1587	127.03***	0.283
[Predicted]	397		397		397		396				
NPB	764	34.1	669	29.9	458	20.5	347	15.5	2238	190.82***	0.292
[Predicted]	560		560		559		559				
MLB	290	29.2	238	24.0	236	23.8	228	23.0	992	9.71*	0.099
[predicted]	248		248		248		248				

The number in the second row is the expected number of players obtained using the chi-square test

*n* number of players, *X*^*2*^ chi-squared value

*p < 0.05; ***p < 0.001

**Table 2:** Odds ratio and 95% confidence interval for each group

	Q1 vs. Q4	Q2 vs. Q4	Q3 vs. Q4	1st vs. 2nd
Elementary school	1.49 (1.25–1.78)	1.3 (1.08–1.56)	1.22 (1.01–1.47)	1.25 (1.10–1.41)
Junior high school	1.71 (1.50–1.95)	1.47 (1.28–1.68)	1.21 (1.05–1.39)	1.44 (1.31–1.58)
High school	2.06 (1.77–2.39)	1.88 (1.62–2.19)	1.24 (1.05–1.46)	1.76 (1.59–1.95)
NPB	2.20 (1.94–2.50)	1.93 (1.70–2.20)	1.32 (1.15–1.52)	1.78 (1.63–1.94)
MLB	1.27 (1.07–1.51)	1.04 (0.87–1.25)	1.04 (0.87–1.25)	1.14 (1.01–1.29)

*1st* the first semester (Q1 and Q2), *2nd* the second semester (Q3 and Q4)

Table 3 shows the results of chi-square tests, dividing junior players into recreational and elite players in each category. Significant RAEs were observed in the recreational junior high and high school players (*p* < 0.001, respectively), and in the elite elementary, junior high, and high school players (*p* < 0.001, respectively). The effect sizes of the RAEs became larger with increasing grade in the recreational players, whereas those became smaller with decreasing grade in the elite players. Table 4 shows OR and 95% confidence intervals for each category in the junior players. The larger effects of OR were observed in Q1, Q2, and Q3 vs. Q4 (OR = 7.10, 4.70, and 2.90, respectively), and 1st and 2nd semesters (OR = 3.03) among elite elementary school players; in Q1 vs. Q4 (OR = 2.54), and 1st and 2nd semesters (OR = 1.92) among elite junior high school players; and in Q1, and Q2 vs. Q4 (OR = 2.40, and 2.24, respectively), and 1st and 2nd semesters (OR = 2.02) among elite high school players. On the other hand, the large effects of OR were not observed in the recreational junior players.

**Table 3:** Distribution of junior players divided into recreational and elite categories

	Q1		Q2		Q3		Q4		Total	*X* ^2^	Effect size
	*n*	(%)	*n*	(%)	*n*	(%)	*n*	(%)	*n*		*w*
Recreational elementary school	227	26.9	212	25.1	215	25.5	190	22.5	844	3.38	0.063
[predicted]	211		211		211		211				
Recreational junior high school	420	29.5	392	27.5	335	23.5	279	19.6	1426	32.44***	0.151
[predicted]	357		357		356		356				
Recreational high school	267	32.2	239	28.8	176	21.2	148	17.8	830	42.81***	0.227
[predicted]	208		208		207		207				
Elite elementary school	71	45.2	47	29.9	29	18.5	10	6.4	157	49.79***	0.563
[predicted]	40		39		39		39				
Elite junior high school	175	38.9	121	26.9	85	18.9	69	15.3	450	57.60***	0.358
[predicted]	113		113		112		112				
Elite high school	262	34.6	244	32.2	142	18.8	109	14.4	757	88.84***	0.343
[predicted]	190		189		189		189				

The number in the second row is the expected number of players obtained using the chi-square test

*n* number of players, *X*^*2*^ chi-squared value

***p < 0.001

**Table 4:** Odds ratio and 95% confidence interval for each group

	Q1 vs. Q4	Q2 vs. Q4	Q3 vs. Q4	1st vs. 2nd
Recreational elementary school	1.19 (0.98–1.44)	1.12 (0.92–1.36)	1.13 (0.93–1.37)	1.08 (0.94–1.24)
Recreational junior high school	1.51 (1.30–1.76)	1.41 (1.21–1.64)	1.20 (1.13–1.41)	1.32 (1.19–1.47)
Recreational high school	1.80 (1.47–2.20)	1.61 (1.31–1.98)	1.19 (0.96–1.48)	1.56 (1.36–1.79)
Elite elementary school	7.10 (3.66–13.77)	4.70 (2.38–9.30)	2.90 (1.41–5.95)	3.03 (2.11–4.35)
Elite junior high school	2.54 (1.92–3.36)	1.75 (1.30–2.35)	1.23 (0.90–1.69)	1.92 (1.60–2.30)
Elite high school	2.40 (1.92–3.00)	2.24 (1.79–2.81)	1.30 (1.01–1.67)	2.02 (1.74–2.35)

Table 5 shows data on the height and weight in each group. The Kruskal-Wallis test showed significant differences in height among Q1–Q4 in the recreational elementary school players (*p* < 0.001, *r* = 0.15). The Mann-Whitney *U* test revealed that the elite elementary school players were significantly taller than the recreational elementary school players in Q1, Q2, Q3, and Q4 (*p* < 0.001, *r* = 0.53; *p* < 0.001, *r* = 0.42; p < 0.001, r = 0.42; *p* < 0.001, *r* = 0.27, respectively). The Kruskal-Wallis test showed significant differences in weight among Q1–Q4 in the recreational elementary school players (*p* < 0.01, *r* = 0.13). The Mann-Whitney *U* test revealed that the elite elementary school players were significantly heavier than the recreational elementary school players in Q1, Q2, Q3, and Q4 (*p* < 0.001, *r* = 0.52; *p* < 0.001, *r* = 0.38; *p* < 0.001, *r* = 0.37; *p* < 0.01, *r* = 0.21, respectively).

**Table 5:** Anthropometric data in each category

						Kruskal-Wallis test
		Q1	Q2	Q3	Q4	(Differences among Q1–Q4)
Recreational elementary school	Height (cm)	141.9	142.1	140.7	138.6	*p* < 0.001
	Weight (kg)	35.5	35.1	34.1	32.9	*p* < 0.01
Elite elementary school	Height (cm)	157.1***	156.9***	154.7***	153.6***	
	Weight (kg)	50.4***	48.4***	50.1***	40.6**	
Recreational junior high school	Height (cm)	163.1	161.6	160.5	157.3	*p* < 0.001
	Weight (kg)	53.8	52.4	51.4	48.1	*p* < 0.001
Elite junior high school	Height (cm)	170.1***	167.7*	164.5*	161.0*	*p* < 0.001
	Weight (kg)	62.4***	60.4***	56.1**	52.9**	*p* < 0.001
Recreational high school	Height (cm)	172.3	171.3	172.1	170.9	
	Weight (kg)	68.2	68.4	67.9	65.9	*p* < 0.01
Elite high school	Height (cm)	173.0	173.8***	172.1	171.0	*p* < 0.001
	Weight (kg)	71.2***	70.6**	69.5 *	69.9**	
NPB	Height (cm)	180.4	180.4	179.9	179.7	*p* < 0.05
	Weight (kg)	80.1	80.2	80.4	79.8	
MLB	Height (cm)	189.9	190.0	189.5	190.5	
	Weight (kg)	94.3	94.6	95.0	95.1	

Data are expressed as the mean. Significant differences between recreational and elite players *p < 0.05, **p < 0.01, and ***p < 0.001

The Kruskal-Wallis test showed significant differences in height among Q1–Q4 in the recreational and elite junior high school players (*p* < 0.001, *r* = 0.22; *p* < 0.001, *r* = 0.35, respectively). The Mann-Whitney *U* test revealed that the elite junior high school players height were significantly taller than the recreational junior high school players in Q1, Q2, Q3, and Q4 (*p* < 0.001, *r* = 0.40; *p* < 0.001, *r* = 0.31; *p* < 0.05, *r* = 0.12; *p* < 0.05, *r* = 0.14, respectively). The Kruskal-Wallis test showed significant differences in weight among Q1–Q4 in the recreational and elite junior high school players (*p* < 0.001, *r* = 0.20; *p* < 0.001, r = 0.31, respectively). The Mann-Whitney *U* test revealed that the elite junior high school players were significantly heavier than the recreational junior high school players in Q1, Q2, Q3, and Q4 (*p* < 0.001, *r* = 0.38; *p* < 0.001, *r* = 0.32; *p* < 0.01, *r* = 0.14; *p* < 0.01, *r* = 0.16, respectively).

The Kruskal-Wallis test showed significant differences in height among Q1–Q4 in the elite high school players (*p* < 0.001, *r* = 0.19). The Mann-Whitney *U* test revealed that the elite high school players were significantly taller than the recreational high school players in Q2 (*p* < 0.001, *r* = 0.20). The Kruskal-Wallis test showed significant differences in weight among Q1–Q4 in the recreational high school players (*p* < 0.01, *r* = 0.12). The Mann-Whitney *U* test revealed that the elite high school players were significantly heavier than the recreational high school players in Q1, Q2, Q3, and Q4 (*p* < 0.001, *r* = 0.20; *p* < 0.01, *r* = 0.14; *p* < 0.05, *r* = 0.14; *p* < 0.01, r = 0.19, respectively).

The Kruskal-Wallis test showed significant differences in height among Q1–Q4 in the NPB players (*p* < 0.05, *r* = 0.07). No significant differences in height or weight were observed among Q1–Q4 in the MLB players.

## Discussion

The present study showed characteristics of RAEs in Japanese baseball players, focusing on recreational and elite junior players. Our data with the effect size and OR showed that the skew of RAEs was larger in the elite junior players than in the recreational junior players. Some previous studies reported that the skew of RAEs was related to players’ level and categories [2, 3, 10, 23–25]. In the present study, elite players were defined as players who played in a national school tournament in Japan. They had not been selected by a national team or coach, but had won many games in prefectural tournaments, and participated in national championship games. Relatively older players may have greater opportunities for selection and experience in childhood because they are naturally heavier, taller, stronger, and faster, have greater endurance and are more coordinated than younger players during childhood [15, 28, 29], all of which translate into performance advantages in most sports. This has been often described as the “maturation-selection hypothesis” (see a review, [15]). Recently, Romann and colleagues reported that RAEs in male youth athletes were more marked among national to international level players than among regional to national level players in Olympic sports [25]. Our data support these previous findings.

We showed significant RAEs in recreational junior high school and high school players, but not in elementary school players. The effect size became larger with increasing grade (i.e., 0.063 in elementary school players, 0.151 in junior high school players, and 0.227 in high school players). There are two possible hypotheses to explain this finding. The first hypothesis is that relatively younger players gradually drop out from playing baseball even in “recreational” players. Delorme and colleagues [5] reported that the relative age was associated with sport dropout. They showed that relatively younger players in categories of 9–10 years old, 11–12 years old, and 13–14 years old tended to drop out from basketball, compared with relatively older players. This may be supported by the anthropometric data. That is, height and weight values were significantly lower in relatively younger players compared with relatively older players (Table 5). Our previous study, focusing on a general sample of Japanese elementary and junior high school students, also reported a significant RAE for boys aged 7–15 years based on fitness data, involving the 50-m sprint, standing long jump, grip strength, bent-leg sit-ups, sit-and-reach, side steps, 20-m shuttle run, and ball throw [26]. Arrieta and colleagues reported a significant correlation between the RAE and a performance parameter in basketball, especially among male players compared with female players [30]. We hypothesized that relatively younger players had difficulty developing self-confidence in childhood, because of disadvantages regarding their body size and sporting performance. The present study did not directly obtain data on dropping out. However, judging from changes in the effect size with increasing grade, relatively young recreational players may drop out from playing baseball in their junior and high school. In future studies, detailed numbers and factors affecting dropping out from playing baseball should be examined. The second hypothesis is that the number of relatively older children who start to play baseball gradually increases with increasing grade. To the best of our knowledge, no data are available on this topic from the Japan Sports Agency. Further studies are also needed to clarify this hypothesis.

On the other hand, the effect size of elite players became smaller with increasing grade (i.e., 0.563 in elementary school players, 0.358 in junior high school players, 0.343 in high school players, and 0.292 in NPB players). These data indicate that advantages of RAE are expected to become less apparent toward adulthood when the status of biological maturity evens out. Indeed, the effect size among Q1–Q4 was smaller in elite high school players than in elite elementary and junior high school players. This phenomenon has already been confirmed in some sports such as soccer, handball, and swimming [2, 6, 7].

We also have to consider the definition of “elite” players in sports. Anthropometric data in Table 5 show that height and weight values were significantly larger in elite elementary and junior high school players than in recreational elementary and junior high school players, while height and weight are generally related to each other. This suggests that biological maturity occurs earlier in elite elementary and junior high school players than in recreational elementary and junior high school players. An important point regarding our anthropometric data was that the height was almost the same between recreational and elite high school players, indicating that the status of biological maturity evens out in high school. Recently, Müller et al. [24] investigated the influence of the biological maturity status on RAEs in youth skiers, and showed that the peak height velocity as an indicator of the biological maturity status was significantly earlier in national ski racers than in provincial ski racers. We considered that the significant difference in weight between recreational and elite high school players was related to strength and weight training.

We also analyzed the data in NPB and MLB. Our data showed the presence of significant RAEs in NPB and MLB, and the effect size and OR were larger in NPB than in MLB. Grondin and Koren [18] reported that RAEs for baseball were more important in Japan than in the USA because large numbers of Japanese players were born during Q1 (April–June). Our data confirmed this, even after the passing of approximately two decades. We suggest that the popularity of high school baseball could be a factor in the larger RAEs in Japan. There are two annual national high school baseball tournaments in Japan, in spring and summer. These tournaments started in 1915. High school baseball was as popular as professional baseball, and to be able to go to Koushien, where the national high school competition was held, was an honor for all young baseball players and schools. Indeed, our previous study using a historical analysis showed that significant RAEs were observed among players born from the 1910s onwards [20]. Some previous studies attempted to reduce the skew of RAEs [31–33]. For example, Mann and van Ginneken [33] reported that the selection bias was eliminated when scouts watched games knowing that the shirt numbers corresponded to the relative ages of the players. We do not know why the skew of RAEs was smaller in MLB players, but it is likely that the education system for junior baseball players differs between Japan and the USA. Further studies are needed to reduce RAEs in Japanese baseball players.

As limitations of the present study, we extracted data from anthropometric data obtained by a national survey and official websites, rather than direct recording by experimenters. Some previous studies used a radiological examination of the wrist [34, 35], and peak height velocity [24] to assess biological maturity. Thus, the accuracy of the present data may be limited. In addition, height and weight were the only indicators of biological maturity. If other indices were used to assess the maturation, the results and interpretation might be changed. These phenomena also might be specific to Japan, because many activities, sports-related or academic, are based on a unique cutoff date (April 1), which is not the case in other countries. Data on RAEs of junior baseball players should be examined in other countries.

## Conclusion

The characteristics of RAEs differed between recreational and elite junior baseball players. Anthropometric data were also different, depending on birth data, and between recreational and elite baseball players in elementary and junior high school students. These underlying data may be useful to reduce RAEs and the selection bias in junior baseball players in future studies.

## Data Availability Statement

The data that support the findings of this study are available on request from the first author [Y. K.].

## Additional file

Additional file 1: The survey form ( in Japanese). (PDF 123 kb)

## Abbreviations

MLB: Major league baseball
NPB: Nippon Professional Baseball
RAE: Relative age effect
