# Supplementary material for: Characteristics of Relative Age Effects and Anthropometric Data in Japanese Recreational and Elite Male Junior Baseball Players
Source: Sports Med Open. 2018 Nov 30;4:52. doi: 10.1186/s40798-018-0165-9 (PMC6265376; doi:10.1186/s40798-018-0165-9)
Supplement: Supplementary file 1 — The survey form ( in Japanese). (PDF 123 kb) [file 40798_2018_165_MOESM1_ESM.pdf]

野球選手のスポーツ経験に関する調査

この調査は、ジュニアスポーツ選手がより良い環境でスポーツを行えるようにするために必要な資料を収集する目的で実施します。アンケートの結果はすべて機械的・統計的に処理されるため、個人情報は公開されませんし、調査・研究目的以外には使用いたしません。現在・未来のスポーツ選手育成のための調査ですので、ぜひ賛同の上、よろしくご協力ください。

調査にご協力いただける場合には□に✔をしてください

※対象者（選手）：研究の目的および方法についての説明を受け、主旨を十分に理解し協力することに同意します. □

※保護者：研究の目的および方法についての説明を受け、主旨を十分に理解し協力することに同意します. □

1-1. 性別（当てはまる□に✔をしてください）と身長・体重を記入してください.

男性 □　女性 □　身長 [　　] cm　体重 [　　] kg

1-2. 生年月日

西暦 [　　] 年 [　　] 月 [　　] 日　例）2002年5月5日

1-3. 兄弟構成（該当する□に人数を記載してください）

兄 □　姉 □　弟 □　妹 □　双子 □

2-1. 自分のチームでよく守るポジションの□に✔をしてください（複数回答可）.

投手 □　捕手 □　内野手 □　外野手 □

2-2. 競技を開始した時期（野球少年団への入団時期）を記入してください.

競技開始時期　小学 [　　] 年生 [　　] 月

2-3. 現在の所属チームにおける今年度の最もよかった競技成績について当てはまる□に✔をしてください.

全国大会入賞（8位以上） □　全国大会出場 □　都道府県大会入賞（8位以上） □

都道府県大会出場 □　市・区内大会入賞（3位以内） □　該当なし □

2-4. これまでの野球生活および今後について、当てはまる□に✔をしてください.

			どちらともいえない			
（1） チーム練習以外にも練習する	□	………	□	………	□	チームでの練習のみである
（2） 怪我に悩んだことが多かった	□	………	□	………	□	体は丈夫だった
（3） プロ野球選手になりたい	□	………	□	………	□	野球と将来の職業は別に考えている
（4） 指導者に恵まれた	□	………	□	………	□	指導者に恵まれなかった
（5） 練習環境（施設）に恵まれた	□	………	□	………	□	練習環境に恵まれなかった
（6） 仲間に恵まれた	□	………	□	………	□	仲間に恵まれなかった
（7） 保護者のサポートに恵まれた	□	………	□	………	□	保護者のサポートに恵まれなかった
（8） 身体の大きさに恵まれた	□	………	□	………	□	身体は同学年の中で小さいほうだ
（9） 自ら野球（少年団）を始めた	□	………	□	………	□	他者からの勧めで野球を始めた
（10） 中学校でも野球を続けたい	□	………	□	………	□	中学校では他のスポーツを行いたい
（11） 試合に出場することが多い	□	………	□	………	□	試合にはあまり出ていない

以上で調査は終了です。ご協力ありがとうございました.　J
